# The sternum in detail: a review of the anatomy and pathologies of the sternum

**DOI:** 10.1590/0100-3984.2024.0128-en

**Published:** 2025-06-02

**Authors:** Margrit Elis Müller, Lara Carolina Peixoto Quiche, Lucas Daniel Pereira Lopes, Adham do Amaral e Castro, Eduardo Kaiser Ururahy Nunes Fonseca

**Affiliations:** 1 Hospital Israelita Albert Einstein (HIAE), São Paulo, SP, Brazil; 2 Escola Paulista de Medicina da Universidade Federal de São Paulo (EPM-Unifesp), São Paulo, SP, Brazil

**Keywords:** Sternum, Manubrium, Xiphoid bone, X-Rays, Multidetector computed tomography, Magnetic resonance imaging, Esterno, Manúbrio, Processo xifoide, Raios X, Tomografia computadorizada multidetectores, Ressonância magnética

## Abstract

The sternum and the sternoclavicular joints can exhibit a wide range of anatomical variations and serve as sites for numerous diseases, many of which are diagnosed solely through imaging studies. Recognizing these variations and differentiating them from pathological conditions is essential for radiologists, because accurate identification helps prevent misdiagnoses and treatment delays. This study provides a comprehensive review of the sternal anatomy, addressing anatomical variations, as well as mechanical, inflammatory, and traumatic pathologies, discussing their radiographic characteristics across different imaging modalities. Thus, it provides an overview of the key radiological findings.

## Abstract (pt)

O esterno e as articulações esternoclaviculares podem apresentar ampla gama de variações anatômicas e ser sítios de diferentes doenças, muitas das quais diagnosticadas somente por meio de exames de imagem. Reconhecer essas variações e diferenciá-las de condições patológicas é essencial para o radiologista, uma vez que a identificação precisa pode evitar diagnósticos errôneos e atrasos no tratamento. Este estudo revisa a anatomia do esterno, abordando as variações anatômicas, as patologias mecânicas, inflamatórias e traumáticas, e discute suas características radiográficas nas diferentes modalidades de imagem, proporcionando uma revisão dos seus principais achados radiológicos relevantes.

## INTRODUCTION

Anatomical alterations and variations of the sternum are commonly encountered in clinical practice, and adequate recognition of these conditions is essential to avoid mistaken diagnoses. This illustrative article reviews the anatomy and pathologies of the sternum, highlighting the radiological features of the anatomical variations and the main pathologies in the different imaging modalities, with a review of the pertinent radiological findings to improve the understanding and clinical management of these conditions.

## ANATOMY AND IMAGING MODALITIES

The sternum is a flat bone composed of three segments: the manubrium, the body, and the xiphoid process. The manubrium is the widest and most cranial segment^**(^1^)**^, presenting a superior central notch (jugular notch) and two lateral fossae (clavicular notches), articulating laterally with the clavicles (sternoclavicular joints) and with the first two ribs, as well as inferiorly with the body at the manubriosternal joint (sternal angle).

**Figure 1:**
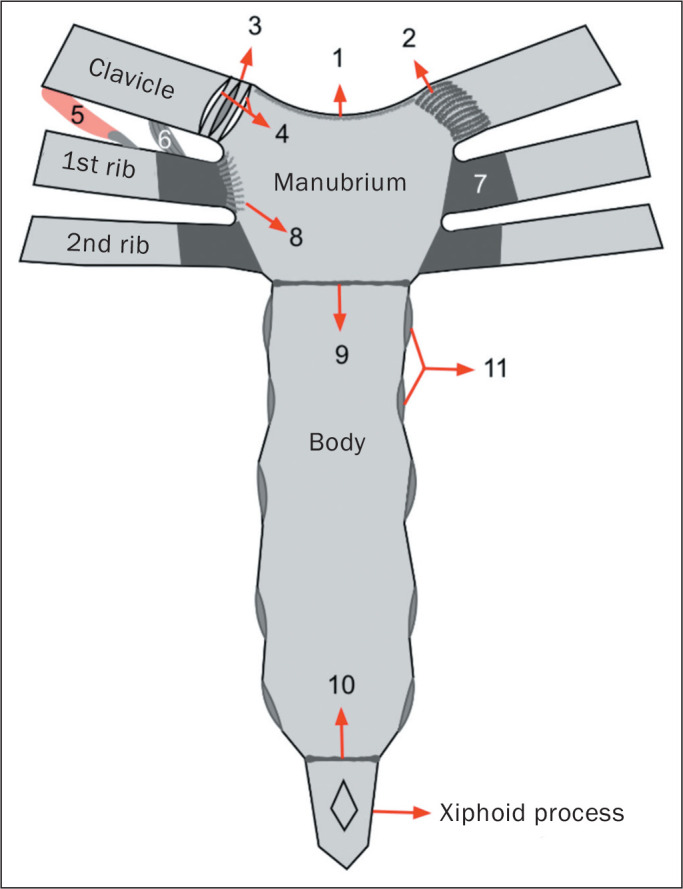
Normal anatomy of the sternum. Schematic drawing demonstrating the three sternal segments (manubrium, body, and xiphoid process), the clavicles, the first two ribs, the interclavicular ligament (1), the anterior sternoclavicular ligament (2), the sternoclavicular intra-articular disc (3), the sternoclavicular joint cavities (4), the subclavius muscle (5), the costoclavicular ligament (6), the costal cartilages (7), the radiated sternocostal ligament (8), the manubriosternal symphysis (9), the xiphisternal symphysis (10), and the articular facets for the ribs (11).

The body of the sternum articulates superiorly with the manubrium and inferiorly with the xiphoid process (xiphisternal joint). The lateral borders join with the second through seventh ribs at the sternocostal joints. The xiphoid process, a thin, elongated bony structure, exhibits considerable anatomic variation in shape and size^**(^1^-^3^)**^, as depicted in Figure 1.

Conventional radiography is still used as the initial examination for evaluating the sternum, especially in cases of trauma. However, because of its ability to avoid image overlap, computed tomography (CT) is the examination of choice for detailed evaluation of bone structures. Magnetic resonance imaging (MRI), for its part, allows a better evaluation of soft tissues and bone marrow edema, which makes it useful for the diagnosis of inflammatory and neoplastic conditions^**(^3^-^5^)**^.

**Figure 2:**
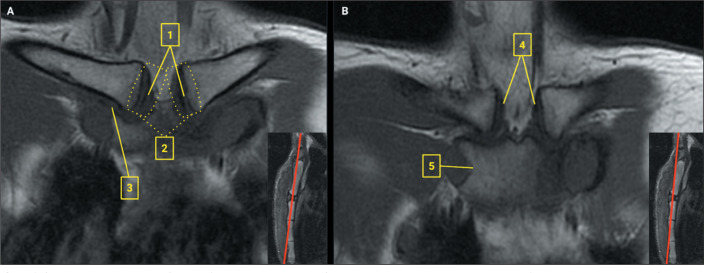
Sternal anatomy on MRI. A: Coronal T1-weighted image without fat saturation showing the intra-articular disc (1), the sternoclavicular joint (2), and the costoclavicular ligament (3). B: Coronal T1-weighted image, positioned more anteriorly in relation to image A, demonstrating the anterior sternoclavicular ligaments (4) and the sternocostal synchondrosis of the first rib (5).

On MRI scans, the sternal anatomy is preferentially evaluated on T1-weighted sequences, whereas pathological changes are best evaluated on fluid-sensitive sequences. As illustrated in Figure 2, the articular surfaces and intra-articular disc can be accurately evaluated in the coronal plane^**(^5^)**^, whereas the costoclavicular ligament can be identified in the sagittal plane; the sternoclavicular joint capsule (in its anterior and posterior aspects) is best visualized in the axial plane, as are the anterior and posterior sternoclavicular ligaments.

## ANATOMICAL VARIATIONS

Anatomical variations of the sternum can be classified didactically into two groups. The first comprises asymptomatic variations, such as sternal foramina, morphological variations of the xiphoid process, and disproportionality of the sternal components. The second comprises deformities associated with the orientation of the sternum in relation to the ribs, such as pectus carinatum and pectus excavatum, which can have aesthetic repercussions or, in some cases, even cause symptoms, requiring surgical correction^**(^6^)**^.

### Sternal foramen

**Figure 3:**
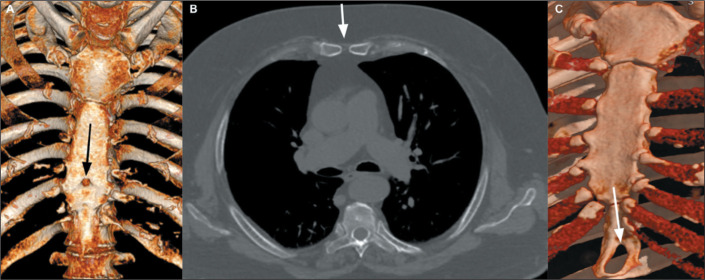
Sternal foramen. A: Three-dimensional reconstruction from a coronal CT scan, showing a foramen in the sternal body (arrow). B: Axial CT scan, with bone window settings, demonstrating the characteristic “bowtie” appearance of the sternal foramen (arrow). C: Three-dimensional reconstruction from a coronal CT scan, showing a foramen in the xiphoid process (arrow).

**Figure 4:**
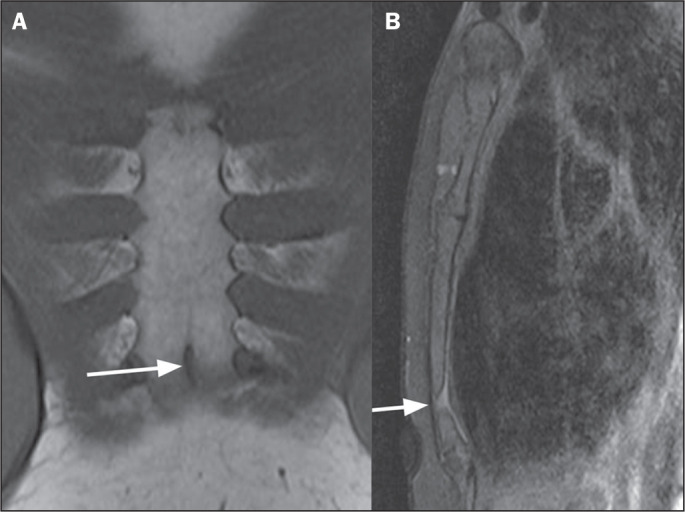
Sternal foramen. A: Coronal T1-weighted MRI of the sternum with fat saturation, showing a foramen in the sternal body (arrow). B: Sagittal T1-weighted MRI of the sternum with fat saturation, showing a foramen in the sternal body (arrow).

A sternal foramen is an anatomical variation of the midline of the sternum, resulting from incomplete fusion of ossification centers during embryonic development, and is identified in approximately 5% of the population^**(^6^)**^. This condition is most frequently seen in the sternal body, although it can also occur in the xiphoid process. On CT, a sternal foramen can mimic an osteolytic lesion. Nevertheless, CT and MRI are both effective methods for its identification. Classically, it has a “bowtie” appearance on axial images (Figure 3). Despite being asymptomatic, a sternal foramen (Figure 4) can increase the risk of complications in patients undergoing acupuncture or invasive procedures, such as sternal bone marrow aspiration, and should be correctly diagnosed to prevent adverse events, such as pneumothorax and cardiac tamponade^**(^6^,^7^)**^.

### Xiphoid process variations

**Figure 5:**
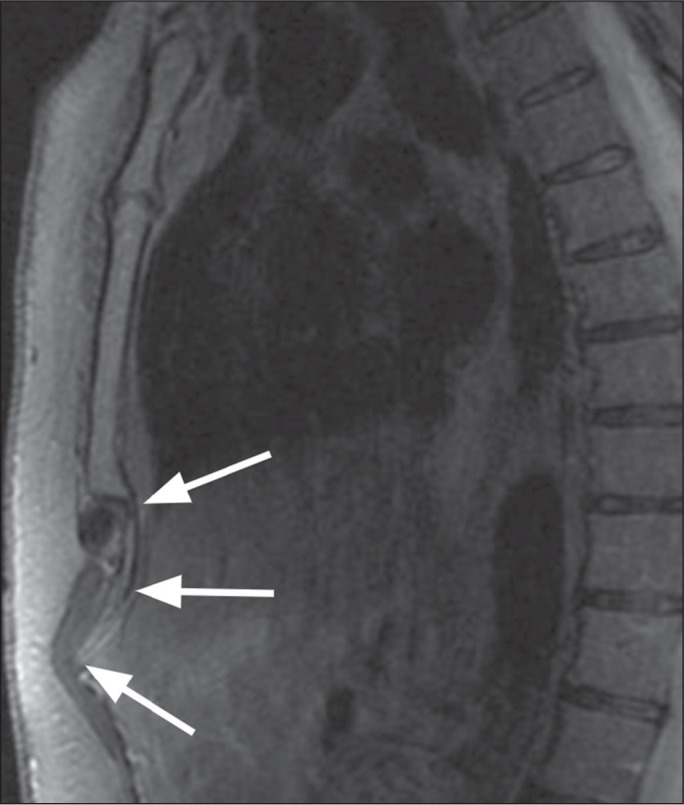
Anterior curvature of the xiphoid process. Sagittal T1-weighted MRI of the sternum, showing a protruding xiphoid process (arrows).

Morphological variations of the xiphoid process are common and represent anatomical variations that are, in most cases, asymptomatic. However, on imaging examinations, some of these variations can cause diagnostic uncertainty. The xiphoid process can vary considerably in shape (being triangular, bifurcated, rounded, or flattened) and size (being short or long). In some cases, it may be more elongated or bifurcated at its extremity, creating an inverted “V” or “Y” appearance^**(^8^,^9^)**^. In addition, some variations may exhibit anterior or posterior curvatures, causing the xiphoid process to project forward or backward. The anterior curvature (Figure 5), in particular, can be palpated externally, causing discomfort, and can, in some cases, be mistaken for a mass in the epigastric region^**(^9^,^10^)**^.

Ossification of the xiphoid process, when the structure converts from cartilaginous tissue to bone, is a common variation that occurs with advancing age. Although that conversion can result in discomfort or pain on palpation, the ossification is not uniform and occurs in a variable manner among individuals. In early development, ossification of the sternum occurs through endochondral ossification, characterized by the formation of ossification centers within the cartilaginous segments. This process begins in the intrauterine period and continues throughout childhood and adolescence, with the fusion of the ossification centers by central bony bridges in the caudocranial direction. The xiphoid process remains predominantly cartilaginous for a longer period and typically ossifies around the age of 40^**(^10^,^11^)**^.

The xiphoid process can present a small central foramen, resulting from a variation in the closure of the bone structure during development (Figure 3C). The method of choice for evaluating the xiphoid process is CT, which allows detailed visualization of its anatomical variations. Multiplanar reconstructions, maximum intensity projections, and volumetric renderings are imaging techniques that facilitate the identification of these variations^**(^9^,^10^)**^.

### Pectus excavatum

Pectus excavatum is the most common congenital deformity of the sternum, occurring in 1 of every 1,000 live births. In this condition, the sternum is displaced posteriorly in relation to the ribs, causing a deviation and axial rotation of the heart to the left, in addition to a decrease in the space occupied by the left lung^**(^12^,^13^)**^.

Pectus excavatum is most commonly noted in childhood, progressing slowly as the child grows. In most cases, young children do not present symptoms, because of their cardiac and pulmonary reserves. However, as the deformity becomes more pronounced and the chest wall becomes more rigid with growth, the ability to perform aerobic physical activities can be limited, thus reducing physical fitness^**(^14^,^15^)**^.

**Figure 6:**
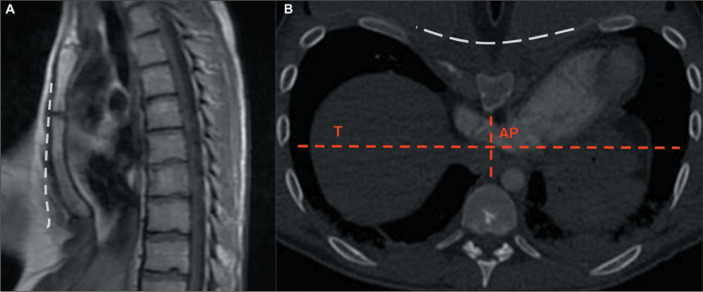
Pectus excavatum. A: Sagittal T1-weighted MRI of the sternum, sagittal plane, with a dashed line representing the normal plane of the sternum. B: Axial CT of the chest, with bone window settings, with a dashed white line representing the normal plane of the sternum. The Haller index, obtained by dividing the transverse diameter of the thorax (T) by its anteroposterior diameter (AP), was 4.6 in this patient.

Although the diagnosis of pectus excavatum is based on clinical findings, the method of choice for its evaluation is CT (Figure 6), because it allows accurate measurement of the degree of cardiac displacement, pulmonary compression, and thoracic asymmetries. The use of CT is also essential for calculating the Haller index, an objective parameter of severity of the condition, obtained by determining the ratio between the largest transverse diameter of the thorax and its smallest anteroposterior diameter. A normal Haller index is 2.56, with a standard deviation of 0.35. However, it is used primarily to classify the severity of pectus excavatum, rather than as a diagnostic criterion. A Haller index greater than 3.25 is suggestive of pectus excavatum, potentially being an indication for surgical correction^**(^2^,^12^,^13^)**^.

The indication for surgical treatment of pectus excavatum is based on the presence of two or more of the following criteria^**(^12^,^14^)**^: a Haller index greater than 3.25; pulmonary function studies showing restrictive or obstructive lung disease; cardiac abnormalities; documented progression of the deformity accompanied by clinical symptoms; failure of a previous Ravitch procedure (surgical removal of the anomalous rib cartilage and remodeling of the sternum); and failure of a previous minimally invasive surgical intervention.

### Pectus carinatum

Pectus carinatum is characterized by anterior displacement of the sternum in relation to the ribs and occurs in 1 in 1,500 live births, being more common in males. Pectus carinatum is usually detected in childhood, especially during periods of peak growth, unlike pectus excavatum, which is often identified at birth. Most patients with pectus carinatum are asymptomatic; when symptoms do occur, they are usually related to localized tenderness in the area of the prominence^**(^14^,^16^)**^.

Pectus carinatum is associated with mitral valve disease. However, to our knowledge, there have been no reports of cardiopulmonary limitation caused by the condition in patients without congenital heart disease. Other associated conditions include Marfan syndrome and scoliosis, suggesting that the etiology of pectus disorders involves a defect in connective tissue developmental^**(^14^-^16^)**^.

**Figure 7:**
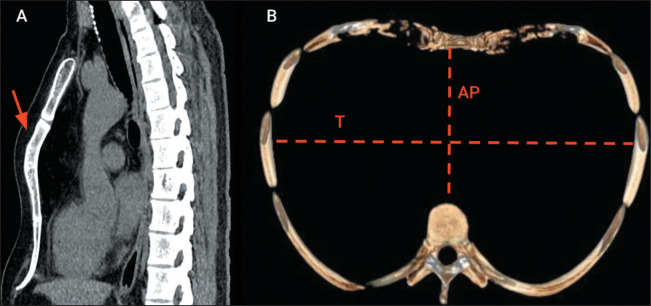
Pectus carinatum. A: Sagittal CT scan, with soft tissue window settings, showing anterior protrusion of the sternum (arrow). B: Three-dimensional reconstruction of an axial CT scan, with bone window settings, showing that the anteroposterior diameter of the thorax (AP) was greater than its transverse diameter (T) in a patient with pectus carinatum (Haller index of 1.48).

In individuals with pectus carinatum, chest X-rays demonstrate anterior protrusion of the sternum and an increase in the anteroposterior diameter of the thorax. As in pectus excavatum, CT is used to calculate the Haller index, and a value between 1.42 and 1.98 is indicative of pectus carinatum. Although the sternum projects ventrally in relation to the chest wall in patients with pectus carinatum, this aspect is typically best appreciated on images obtained in the sagittal plane. On axial images, the sternum may be positioned posteriorly in relation to the rib cage, depending on the level at which the slice is acquired^**(^12^,^17^)**^, as shown in Figure 7.

## DEGENERATIVE AND INFLAMMATORY PATHOLOGIES

The main arthropathies of the sternum include septic arthritis, osteoarthritis, rheumatoid arthritis, ankylosing spondylitis, psoriasis, crystal deposition arthritis, and Tietze syndrome. The examinations of choice for evaluating inflammatory arthropathies of the sternal joints are CT and MRI. Whereas CT detects bone abnormalities and calcifications, MRI allows visualization of the bone marrow and intra-articular structures, including the cartilage and articular disc, as well as the adjacent soft tissues^**(^2^,^18^)**^.

**Figure 8:**
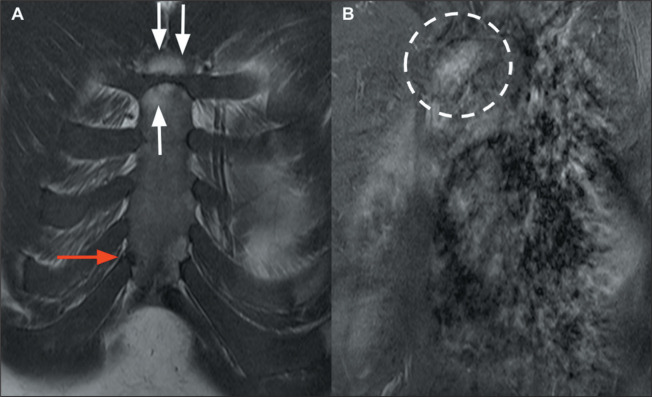
SAPHO syndrome. A: Coronal T1-weighted MRI of the sternum, showing costochondritis of the right sixth sternocostal joint (red arrow) and chronic manubriosternal arthropathy with bony irregularities and fatty replacement of its margins (white arrows). B: Gadolinium contrast-enhanced sagittal T1-weighted MRI of the sternum, showing enhancement related to right sternoclavicular inflammatory arthropathy (dashed circle).

Synovitis, acne, pustulosis, hyperostosis, and osteitis (SAPHO) syndrome involves a broad spectrum of neutrophilic dermatoses associated with aseptic osteoarticular lesions and is classically associated with sternal arthropathy (Figure 8). The site most often affected is the sternoclavicular joint; on X-rays and CT scans, the characteristic findings are hyperostosis, osteosclerosis, joint erosion, and ankylosis^**(^19^)**^.

If there is an active lesion in a patient with SAPHO syndrome, T2-weighted MRI with fat saturation shows bone marrow edema, a characteristic that helps differentiate it from chronic lesions. Bone scintigraphy with technetium may show the “bull’s head” sign, characterized by increased radiotracer uptake in the manubrium and both sternoclavicular joints, which is highly specific for the diagnosis of SAPHO syndrome and can preclude the need for biopsy^**(^2^,^19^)**^.

**Figure 9:**
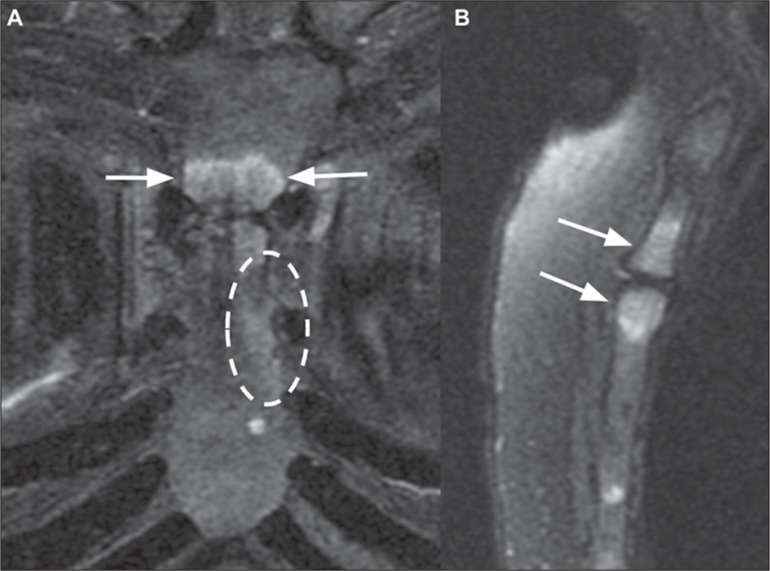
Tietze syndrome. A: Coronal T2-weighted MRI with fat suppression, showing bone edema in the third and fourth sternocostal joints (dashed ellipse) and manubriosternal arthritis (arrows). B: Sagittal T2-weighted MRI with fat suppression, showing manubriosternal arthritis (arrows).

Tietze syndrome is characterized by painful, nonsuppurative swelling of the upper costosternal region, of unknown etiology and pathogenesis. The examination of choice for the diagnosis of the syndrome is MRI, on which typical findings include thickening/swelling of the affected cartilage and edema of the subchondral bone marrow, as well as intense gadolinium uptake in areas of cartilaginous thickening and adjacent tissues (Figure 9). It is essential to differentiate Tietze syndrome from other conditions, such as costochondritis, which does not present with bone edema, and seronegative rheumatic diseases that can also affect the anterior chest wall but present with systemic symptoms and biochemical changes^**(^20^,^21^)**^.

## TRAUMA

Sternal fractures occur primarily in cases of high-energy trauma, such as those resulting from motor vehicle accidents. The mechanism of injury can be direct, such as frontal impact to the chest, or indirect, such as hyperflexion of the thoracic spine, resulting in compression fractures. In children, sternal fractures occur with less intense traumatic impacts because of greater flexibility and lower bone density^**(^22^-^24^)**^.

**Figure 10:**
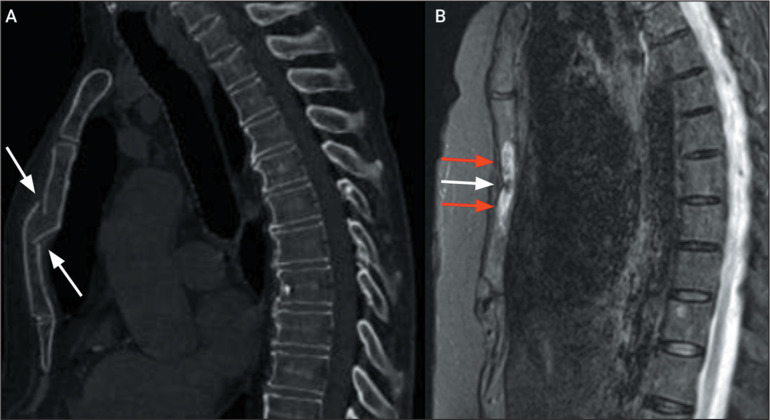
Acute sternal fracture. A: Sagittal CT scan of the chest, with bone window settings, showing a fracture with anterior misalignment (arrows) in the sternal body. B: Sagittal T2-weighted MRI of the sternum (of the same patient), with fat saturation, showing the fracture of the sternal body (white arrow) with impaction and bone edema (red arrows).

Although fractures can occur in any segment of the sternum, they most commonly affect the body (Figure 10). The importance of these injuries lies in the high frequency of associated complications, such as pulmonary contusion, cardiac trauma, and rib fractures, as well as fractures of the cervical, thoracic, or lumbar spine. In addition to trauma, pathological conditions, such as osteoporosis, increase the predisposition to sternal fractures, even in cases of low-energy trauma. Pathological fractures of the sternum, such as those caused by bone metastases, differ from traumatic fractures in that they generally present greater deformity and slower healing^**(^23^-^25^)**^.

Although lateral chest X-ray is effective in detecting most sternal fractures, it does not always reveal intrathoracic injuries. The imaging modality of choice is CT because three-dimensional analysis allows a detailed assessment of fracture patterns, facilitating the identification of the most common morphologies, the diagnosis of associated injuries, and therapeutic planning^**(^26^)**^.

Sternoclavicular dislocation is a rare but potentially serious injury to the joint that connects the manubrium to the medial end of the clavicle. That joint is stabilized by the anterior and posterior sternoclavicular ligaments, together with the interclavicular and costoclavicular ligaments. The most common mechanism of injury involves lateral impact to the shoulder, which generates a lateral compressive force and displaces the clavicle medially. In children and adolescents, medial clavicular physeal separation occurs more frequently than true dislocation because of skeletal immaturity and an unfused physis ^**(^27^,^28^)**^.

Sternoclavicular dislocations are classified as anterior and posterior, according to the direction of displacement of the medial end of the clavicle. Anterior dislocations are more common and usually occur due to direct trauma to the anterior aspect of the shoulder, forcing the clavicle medially and posteriorly. Posterior dislocations, which are less common, are more serious because they can compress vital structures in the mediastinum, such as blood vessels, the trachea, and the esophagus^**(^28^,^29^)**^.

The diagnosis of sternoclavicular dislocations is complex and often requires CT to accurately assess the extent and direction of the dislocation and to differentiate between dislocations and fractures. Treatment depends on the direction of the dislocation and on the associated complications. Anterior dislocations can usually be treated with closed reduction and immobilization, whereas posterior dislocations often require open reduction because of the risk of mediastinal compression^**(^28^)**^.

## INFECTIONS

**Figure 11:**
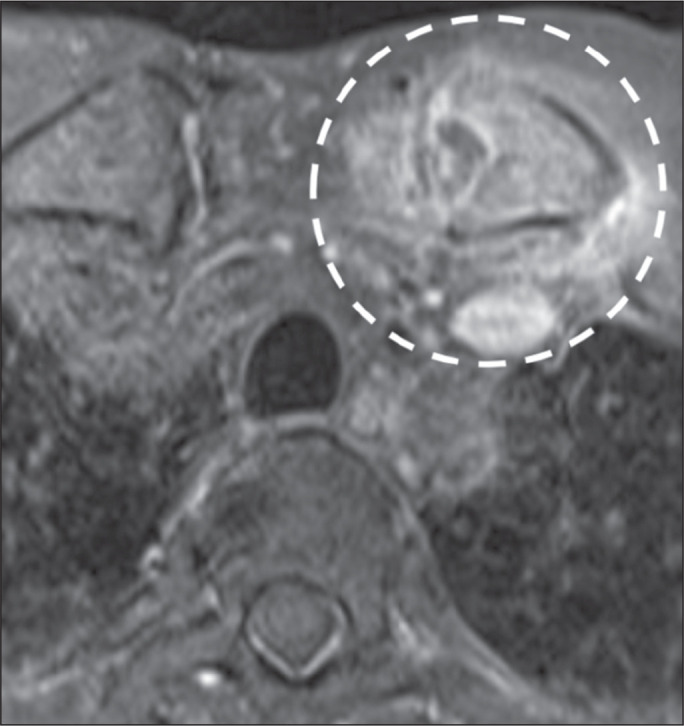
Septic arthritis. Gadolinium contrast-enhanced axial T1-weighted MRI with fat saturation, showing septic arthritis of the left sternoclavicular joint, with joint effusion, synovitis, and periarthritis. Note the enhancement of bone and adjacent soft tissue planes (dashed circle).

Acute septic arthritis of the sternoclavicular joint is a rare infectious condition, typically being monoarticular and having an insidious onset (Figure 11). The most common pathogens are *Staphylococcus aureus* and *Pseudomonas aeruginosa*. Infection can occur by hematogenous spread, especially in individuals with risk factors such as intravenous drug use and diabetes mellitus. The potential complications are serious, including mediastinitis, superior vena cava syndrome, and septic shock^**(^30^)**^.

It has been demonstrated that MRI is effective in identifying capsular distension, bone edema, joint effusion, and inflammatory changes in adjacent soft tissues, all of which are more prominent in infectious arthritis than in conditions such as spondyloarthritis. In addition, CT can be used to identify destruction of the articular surface, increased joint space, and collections in the chest wall ot mediastinum^**(^30^,^31^)**^.

Sternal osteomyelitis is a rare condition, often seen in patients with a history of intravenous drug abuse and in those with immunodeficiency states. The primary form usually results from hematogenous spread of infection. Secondary osteomyelitis is more common, arising as a complication of surgical procedures, commonly associated with the dehiscence of metal sutures. The main risk factors include diabetes mellitus, obesity, prolonged use of corticosteroids, and previous infections. The pathogenesis involves direct contamination of the sternal bone during surgery or the spread of superficial infections to the underlying bone, often being caused by *S. aureus* or *S. epidermidis*^**(^32^-^34^)**^.

**Figure 12:**
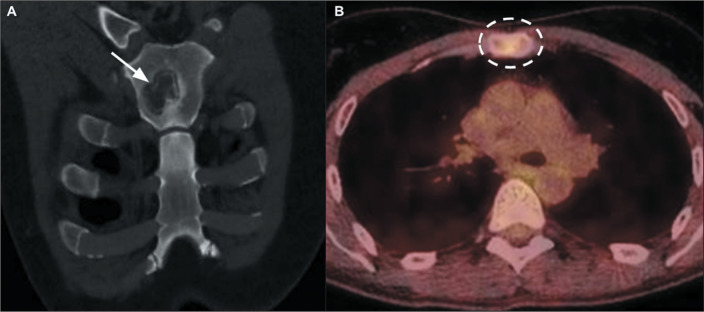
Osteomyelitis of the sternum. A: Coronal CT scan of the chest, with bone window settings, showing a lytic bone lesion (arrow). B: Axial PET/CT showing increased fluorodeoxyglucose uptake (dashed ellipse) in the same patient. The biopsy and culture results were consistent with a diagnosis of sporotrichosis.

**Figure 13:**
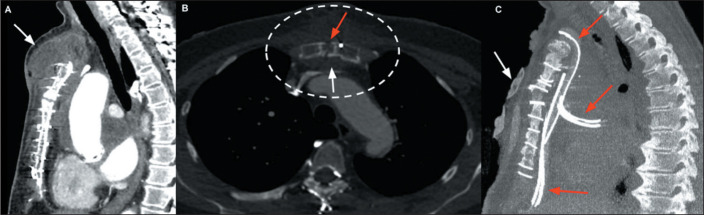
Secondary sternal osteomyelitis with a collection in the anterior chest wall. A: Contrast-enhanced sagittal CT of the chest, with mediastinal window settings, acquired three months after surgical repair of an ascending aortic aneurysm, revealing a collection in the anterior chest wall (arrow). B: Contrast-enhanced axial CT of the chest, with bone window settings, showing the same collection in contact with the sternal sutures (dashed ellipse), where there is a separation between the bony edges (red arrow) at the manubrium, together with cortical irregularities, bone resorption, and increased density of retrosternal adipose tissue (white arrow). C: Contrast-enhanced sagittal CT of the chest, with maximum intensity projection reconstruction, obtained after a new debridement surgery, with a vacuum-assisted closure device (white arrow) and mediastinal drains (red arrows). Cultures confirmed infection with *S. epidermidis*.

The extent of infection can be evaluated by CT, as can abscesses and bony sequestra, whereas MRI is useful in differentiating osteomyelitis from other inflammatory conditions. Positron emission tomography/CT (PET/CT) can show increased fluorodeoxyglucose uptake by the lesion (Figure 12). Treatment of sternal osteomyelitis usually involves targeted antibiotics, as determined by culture results. Surgical interventions, such as sternal debridement or resection, can be necessary in cases of extensive infection or failure of conservative treatment. In addition, vacuum-assisted closure therapy can be used in order to prevent chronic osteomyelitis in deep postoperative infections^**(^33^,^34^)**^, as depicted in Figure 13.

## TUMORS

Most sternal neoplasms are metastases, primary tumors being relatively rare at this site. However, when they occur, primary sternal neoplasms are highly likely to be malignant. Therefore, when a new mass is identified in the sternum, whether primary or secondary, it is recommended to consider it malignant until proven otherwise^**(^2^,^35^)**^.

Sternal metastases occur by direct infiltration from adjacent organs or by hematogenous dissemination, being most common in patients with breast, lung, or thyroid cancer, as well as in those with lymphoma. On CT, sternal metastases can present lytic or sclerotic characteristics, depending on the origin of the primary tumor (as in cases of multiple myeloma and cases of breast or prostate cancer, respectively). On MRI, metastases present a hypointense signal on T1-weighted images and a hyperintense signal on T2-weighted images. Imaging modalities such as PET/CT and bone scintigraphy are also used in order to identify bone metastases, aiding in the assessment of the extent of metastatic disease. Diagnostic confirmation often requires biopsy^**(^36^,^37^)**^.

**Figure 14:**
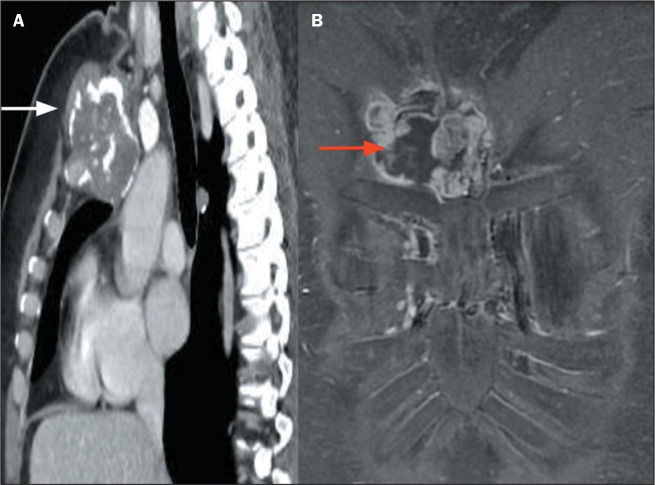
Chondrosarcoma. A: Reconstruction of a contrast-enhanced sagittal CT scan of the chest, with mediastinal window settings, showing a bone lesion with aggressive characteristics in the sternum (arrow) and areas of calcification with a “ring-and-arc” pattern. B: Gadolinium contrast-enhanced coronal T1-weighted MRI of the same patient, with fat saturation, showing an area of necrosis within the lesion (arrow). Biopsy confirmed that this was a chondrosarcoma.

**Figure 15:**
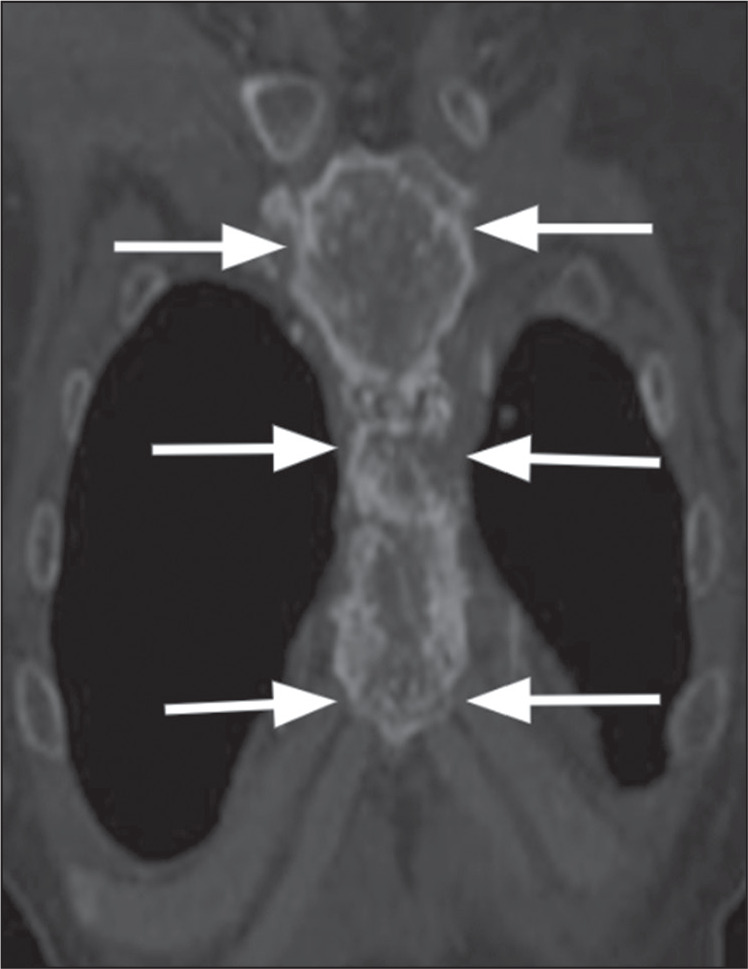
Hemangioendothelioma. Reconstruction of a coronal CT scan of the chest CT, showing aggressive lytic lesions throughout the body of the sternum and manubrium, with areas of rupture of the cortical bone (arrows). Histopathological examination confirmed the diagnosis of hemangioendothelioma.

Primary malignant tumors involving the sternum have distinct features that suggest specific diagnoses. Mineralization of the bone matrix is seen in chondrosarcomas and osteosarcomas. In multiple myeloma, there are lytic, expansile lesions in the sternum and vertebrae. On CT, a chondrosarcoma often presents as a well-defined, lobulated soft tissue mass with areas of dense calcification in the chondroid matrix, which can present as a “ring-and-arc” pattern of calcification on imaging (Figure 14). Hemangioendothelioma is a rare vascular tumor that can present as a painful mass and is often confused with other malignant bone lesions. On CT, it typically presents as a lytic lesion with poorly defined margins that enhances after contrast administration^**(^36^-^38^)**^, as illustrated in Figure 15.

**Figure 16:**
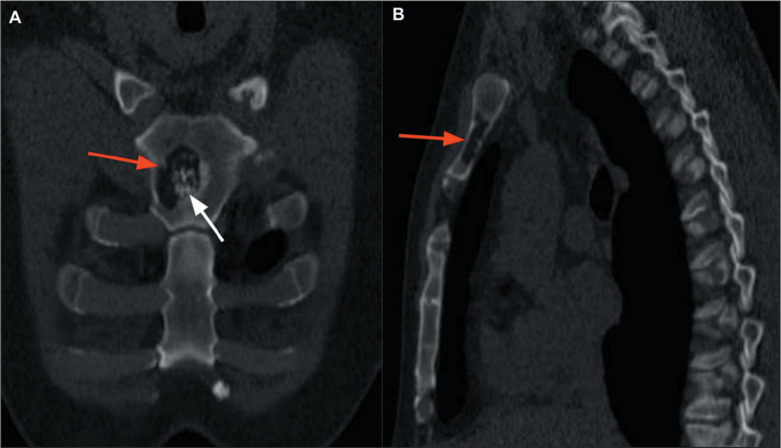
Enchondroma. A,B: Chest CT, with bone window settings, in coronal and sagittal reconstructions, respectively, showing a sternal lytic bone lesion (red arrows), with calcifications characteristic of a chondral lineage lesion (white arrow), without signs of aggressiveness.

Benign primary tumors of the sternum, which are rarer than malignant ones, have specific clinical and radiological characteristics. Among the most common are enchondromas, osteochondromas, osteoid osteomas, and aneurysmal bone cysts. Enchondromas are benign cartilage tumors that develop within the bone (intramedullary tumors). In general, they are asymptomatic and are discovered as incidental findings on imaging examinations. On CT, an enchondroma manifests as a lytic lesion with areas of calcification (Figure 16). However, the diagnostic differentiation between an enchondroma and a low-grade chondrosarcoma is challenging because of the overlap between the imaging findings and the pathology findings^**(^2^,^39^)**^.

The differential diagnosis between enchondroma and chondrosarcoma requires the integration of all available clinical and radiological information, such as patient age, the presence of pain, the history of growth of the lesion, the size/location of the lesion, and the imaging findings. The pathology study also has limitations, because the heterogeneity of a cartilaginous tumor implies that biopsy sampling might not adequately represent significant portions of the lesion^**(^40^)**^.

## POSTOPERATIVE CONDITIONS

**Figure 17:**
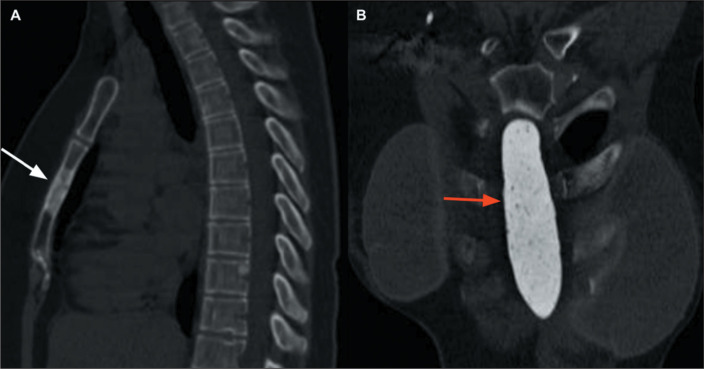
Sternal metastasis of breast carcinoma. A: Sagittal CT of the chest, with bone window settings, showing a heterogeneous, sclerotic lesion in the sternal body (arrow), with suspected secondary neoplastic involvement, in a patient with metastatic breast adenocarcinoma. B: Coronal CT of the chest (of the same patient), after surgical resection of the lesion (metastasectomy) with placement of a sternal body prosthesis (arrow).

The sternum is manipulated in thoracotomy procedures, as it is during the surgical correction of misaligned fractures and resection of lesions in the region. In postoperative imaging examinations, it is essential to evaluate the alignment of the bone structures, consolidation, the persistence of lesions, and the integrity of the osteosynthesis material. Although radical resection of the sternum is rare, when necessary, it can require the placement of prosthetic material^**(^35^,^41^,^42^)**^, as shown in Figure 17.

The most common postoperative complications, which should be distinguished from expected postoperative changes, include dehiscence, pseudarthrosis, secondary osteomyelitis, and mediastinitis. Normal findings after a sternotomy include callus formation, minor displacements, and impactions. Although consolidation typically occurs within six to eight weeks, complete recovery can take three to six months, depending on factors such as age, the presence of diseases such as diabetes, and the use of corticosteroids, which can delay healing^**(^42^,^43^)**^.

Mediastinitis is a deep infection involving the mediastinum that is associated with high rates of morbidity and mortality. The pathophysiology involves bacterial contamination during surgery or hematogenous spread. Imaging findings on CT include gas in the mediastinum, fluid collections, soft tissue thickening, and sternal nonunion^**(^42^,^44^)**^.

**Figure 18:**
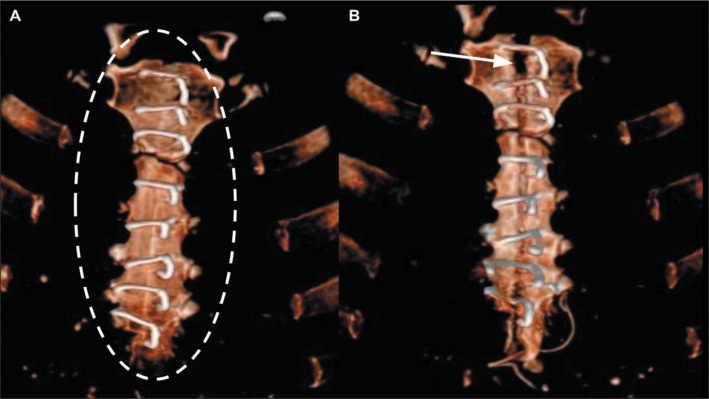
Sternal closure with steel sutures after median sternotomy. A: Three-dimensional reconstruction of a coronal CT scan of the chest, showing the steel sutures (dashed ellipse) eight days after cardiac surgery, with well-approximated bone edges. B: Three-dimensional reconstruction of a coronal CT scan of the chest of the same patient six weeks after surgery, revealing dehiscence of the sternal suture at the central manubrium, with separation of the bone edges (arrow) resulting from a fluid collection in the anterior chest wall.

Sternal dehiscence is defined as the separation of the sternal edges after surgery (Figure 18), whereas sternal nonunion is characterized by failure of bone consolidation due to inadequate healing. Although dehiscence is often associated with infections, it can also result from mechanical factors, such as excessive tension^**(^35^,^42^)**^.

On CT, sternal dehiscence is characterized by a sternal gap greater than 3 mm. Postoperative follow-up by imaging is essential because radiographic changes, such as displacement, rotation or rupture of the sternal cords, usually precede the clinical diagnosis of sternal dehiscence by an average of three days. In sternal nonunion, MRI can reveal signal changes in the adjacent bone marrow, as well as the presence of fluid in the fracture space^**(^40^,^42^,^45^)**^.

## CONCLUSION

It is essential that radiologists recognize the anatomical variations of the sternum and differentiate them from the main sternal pathologies to avoid misinterpreting benign findings, thus providing accurate diagnoses. We hope that this illustrative essay of the broad spectrum of diseases affecting the sternum will help our colleagues to detect those diseases correctly on various imaging modalities, thus enabling the appropriate treatment of patients.
